# Vaginal health and hygiene practices and product use in Canada: a national cross-sectional survey

**DOI:** 10.1186/s12905-018-0543-y

**Published:** 2018-03-23

**Authors:** Sara E. Crann, Shannon Cunningham, Arianne Albert, Deborah M. Money, Kieran C. O’Doherty

**Affiliations:** 1 Department of Psychology, University of Guelph, 50 Stone Road E, Guelph, Ontario N1G 2W1 Canada; 2 Department of Medicine, University of Alberta, 36 Lester Cres, St. Albert, Alberta T8N 2C1 Canada; 3 Women’s Health Research Institute, BC Women’s Hospital and Health Centre, Vancouver, British Columbia V6H 3N1 Canada; 4 Department of Obstetrics and Gynecology, Faculty of Medicine, University of British Columbia, 2329 West Mall Road, Vancouver, British Columbia V6T 1Z4 Canada

**Keywords:** Microbiome, Women’s health, Vaginal health, Hygiene products, Vaginal hygiene, Vaginal practices

## Abstract

**Background:**

The vaginal microbiome influences quality of life and health. The composition of vaginal microbiota can be affected by various health behaviors, such as vaginal douching. The purpose of this study was to examine the types and prevalence of diverse vaginal/genital health and hygiene behaviors among participants living in Canada and to examine associations between behavioral practices and adverse gynecological health conditions.

**Method:**

An anonymous online survey, available in English and French, was distributed across Canada. The sample consisted of 1435 respondents, 18 years or older, living in Canada.

**Results:**

Respondents reported engaging in diverse vaginal/genital health and hygiene behavioral practices, including the use of commercially manufactured products and homemade and naturopathic products and practices. Over 95% of respondents reported using at least one product in or around the vaginal area. Common products and practices included vaginal/genital moisturizers, anti-itch creams, feminine wipes, washes, suppositories, sprays, powders, and waxing and shaving pubic hair. The majority of the sample (80%) reported experiencing one or more adverse vaginal/genital symptom in their lifetime. Participants who had used any vaginal/genital product(s) had approximately three times higher odds of reporting an adverse health condition. Several notable associations between specific vaginal/genital health and hygiene products and adverse health conditions were identified.

**Conclusions:**

This study is the first of its kind to identify the range and prevalence of vaginal/genital health and hygiene behaviors in Canada. Despite a lack of credible information about the impact of these behaviors on women’s health, the use of commercially manufactured and homemade products for vaginal/genital health and hygiene is common. Future research can extend the current exploratory study by identifying causal relationships between vaginal/genital health and hygiene behaviors and changes to the vaginal microbiome.

## Background

The vaginal microbiome (the microbial community in the vagina) influences quality of life, defends against pathogens, and influences fertility and reproductive success [1–3]. Disruptions in the balance of the microbial ecosystem can result in profound health consequences. Current microbiome research is working to determine the microbes that characterize a healthy microbiome in order to link particular microbial profiles with adverse gynecological and obstetrical outcomes [2–8]. The composition of vaginal microbiota can be affected by various health behaviors such as antibiotic use, sexual activity, and behavioral interventions such as douching and birth control methods [9, 10]. Previous research on vaginal health behaviors has focused primarily on vaginal douching. Reports of douching prevalence vary, but the US Centre for Disease Control and Prevention reports approximately 20% of women between 15 and 44 years of age had douched within the last year [11]. This practice has been linked to adverse obstetrical and gynecological health outcomes, such as pelvic inflammatory disease, reduced fertility, ectopic pregnancy, low-birth rate, pre-term pregnancy, cervical cancer, bacterial vaginosis (BV), and higher risk for acquisition of sexually transmitted pathogens [12–17]. For example, a cross-sectional survey with almost 4000 American women found that among asymptomatic women, the prevalence of BV was significantly higher among those who had douched. However, there was no relationship between BV prevalence and douching among symptomatic women. This study also found a significant association between BV prevalence and the use of feminine cleansing wipes, but no association with sprays [18]. Research examining American women’s vaginal practices beyond douching found that those who douched were more likely to use other commercially available vaginal products such as sprays, wipes, powders, and bubble bath for feminine cleaning [19]. Cross-sectional studies in the US have reported between 42 and 53% of women had used sprays, between 17 and 50% used feminine wipes, between 23 and 46% used anti-itch products, and 2% used deodorant suppositories [20, 21]. The use of products in the vaginal area for cleansing and other purposes has also been documented in a number of African and Asian countries [22, 23].

For the efficacy of medical interventions to be optimized, they need to be applied in the context of knowledge of social and cultural practices that shape relevant health behavior. As microbiome research is progressing, it is important to understand the impact of various vaginal practices on vaginal microbiota and subsequent health outcomes. The purpose of the current research is to develop a comprehensive understanding of the diversity and frequency of vaginal/genital health and hygiene behaviors. This paper reports on findings from a large nation-wide online survey of Canadian participants’ vaginal/genital health and hygiene behaviors and health conditions. The purpose of this paper is to: i) describe the types and prevalence of vaginal/genital symptoms and health conditions; ii) describe the types and prevalence of health and hygiene practices; iii) and identify associations between product use and adverse health conditions. As microbiome research continues to advance our understanding of the connection between vaginal microbiome and human health, our aim was to identify health and hygiene behaviors that may influence the health of the vaginal microbiome. This research is a first step in improving our understanding of the role of human behavior on the composition of the vaginal microbiome.

## Methods

### Survey development

Research ethics approval was obtained from the University of Guelph Research Ethics Board. A comprehensive survey about vaginal/genital health and hygiene practices and product use was initially developed via (1) a review of the academic literature; (2) an internet search for vaginal/genital hygiene products and practices; and (3) cataloguing of vaginal/genital hygiene products available for sale at local drugstores. Five focus groups were conducted over 6 months to obtain feedback on survey clarity, cultural sensitivity, and comprehensiveness. Results from the focus groups were used to inform changes to the survey design and content.

The final version asked questions about frequency of use of various products in and around the vaginal area, including products marketed specifically for use in the vaginal area (i.e., washes, wipes, sprays, powders, deodorants, suppositories, anti-itch creams, moisturizers/lubricants, douches, and menstrual products) and general products (i.e., baby wipes, body creams, baby oil). Use of vaginal/genital health and hygiene products internal to the vagina and external to the vagina were reported at 3 months prior to completing the survey. Respondents could provide information on their motivations for using products in open-ended text boxes. Other questions included history of vaginal/genital symptoms and adverse health condition diagnoses (reported within the 6 months prior to completing the survey), history of various vaginal/genital health and hygiene practices, and sexual health history, among other topics. Text boxes allowed respondents to share specific information about products, practices, and experiences not captured in the response options.

### Recruitment

The anonymous online survey, available in English and French, was launched in October 2012 and was available until May 2014. The survey was open to individuals 18 years of age and older living anywhere in the world, but recruitment targeted a Canadian sample. The study was advertised as “women’s health and hygiene” but eligibility was not based on participant gender identity and as such the survey was not limited to participants who identified as “woman” or “female.” Informed consent was obtained prior to the start of the survey. Advertisements were posted on Canadian city webpages of online classifieds (e.g., Kijiji). Recruitment emails with a request to help disseminate the study were sent to Canadian organizations and groups with a possible interest in the research (e.g., women’s health organizations, sexual and gender diversity groups, older women’s groups, Indigenous women’s groups). Social media (i.e., Facebook, Twitter), including paid Facebook advertising, was used to promote the survey around the country. A participant recruitment firm was hired also to assist in recruiting participants from particular demographics.

### Participant demographics

In total, 1471 individuals completed the survey. An additional 233 individuals started but did not submit the survey. The analysis includes the 1435 participants who were living in Canada at the time they completed the survey. The majority of participants (98.6%) identified as cisgender women, one identified as a transwoman (0.1%), eight identified as transmen (0.6%), and 11 provided no answer (0.8%). See Table 1 for additional participant demographics.

**Table 1:** Participant demographics

Age	*N* (%)
18-25 years	451 (31.4)
26-35 years	305 (21.2)
36-45 years	216 (14.8)
46-55 years	239 (16.7)
56-65 years	149 (10.4)
66-75 years	62 (4.3)
76+ years	16 (1.1)
Sexual Orientation	
Heterosexual	1234 (86)
Lesbian	39 (2.7)
Bisexual	99 (6.9)
Questioning/uncertain	22 (1.5)
Other	37 (2.6)
Ethnic/racial Identity	
White	1282 (89.3)
Aboriginal	48 (3.3)
Black	30 (2.1)
Central/South American	7 (0.5)
South Asian	21 (1.5)
East Asian	24 (1.7)
Chinese	24 (1.7)
Middle Eastern/Arab	19 (1.3)
Other	27 (1.8)
Highest Level of Education	
Less than high school	17 (1.2)
High school diploma or equivalent	251 (17.5)
Some post-secondary	319 (22.2)
Diploma program completed	206 (14.4)
Degree program completed	405 (28.2)
Post-graduate completed	235 (16.4)

Note: Participants could select more than one ethnic/racial identity

### Data analysis

Given the exploratory nature of this study, descriptive analysis focused on frequencies of behaviors and adverse health conditions. Univariate and multivariate logistic regression analyses were used to test associations between product use and adverse health conditions. Associations were estimated using odds ratio and 95% confidence intervals. Due to small cell counts, the internal and external product variables were collapsed into dichotomous product variables and all STIs (trichomoniasis, gonorrhea, chlamydia, genital herpes, genital warts, and syphilis) were collapsed into a single dichotomous variable. Dichotomous composite variables were created for the primary outcome (participant report of any type of adverse health condition) and the primary risk factor (participant report of use of any vaginal/genital health and hygiene product). A univariate logistic regression model was fit to test this relationship. To test for potential confounding demographic factors, a series of univariate logistic regression models were fit to test for a relationship between selected demographic factors of interest (ethnic/racial identity, age, education level, and sexual orientation) and adverse health conditions. Significant demographic variables at the *p* < .05 level were entered into a multivariate logistic regression model to test the relationship between product use and adverse health conditions. Finally, univariate logistic regression models were fit to test the associations between each product and each adverse health condition. Missing data (i.e., the participant did not complete the question) were excluded from analysis. Statistical package SPSS 23.0 [24] was used for the analysis.

## Results

The results are reported in three sections: (1) respondents’ reported vaginal/genital symptoms and health conditions, (2) respondents’ reported vaginal/genital health and hygiene behaviors, and (3) associations between commonly used vaginal/genital health and hygiene products and adverse health conditions.

### Symptoms and health conditions

Vaginal/genital symptoms included itching (74.5%), burning (50.2%), unusual discharge (45.2%), redness (34.9%), irritation/rash (21.3%), swelling (17.9%), and sores (10.7%). Eighty percent reported having experienced at least one symptom ever in their lifetime. Almost 36% reported having ever experienced one to two symptoms, 32.2% reported between three and four symptoms, and 17.8% reported five or more symptoms, with 3.1% reporting experiencing each of the seven symptoms at least once in their lifetime. The median number of symptoms reported was 2.0.

Over half of the sample reported having been clinically diagnosed at least once in their lifetime (“ever diagnosed”) with a yeast infection/candida (54.1%) and a urinary tract infection (UTI) (56.1%). Approximately 12% reported bacterial vaginosis (BV) diagnosis at least once in their lifetime. Diagnosis of a sexually transmitted infection (STI) was reported by a smaller proportion of the sample: HPV (6.0%), trichomoniasis (2.2%), genital warts (6.1%), genital herpes (3.8%), chlamydia (5.6%), gonorrhea (2.2%), and syphilis (0.3%). Approximately 6% of the sample reported having ever been diagnosed with cervical cancer.

### Vaginal/genital health and hygiene product use and practices

Respondents reported using a wide variety of commercially manufactured and homemade health and hygiene products (Table 2) and engaging in diverse health and hygiene practices (Table 3). The analysis presented in this paper excludes menstrual products. Approximately 95% of the sample reported using at least one product in the vaginal/genital area. The number of products used by respondents ranged from 0 to 14 (*M* = 3.80, *SD* = 2.18). Respondents reported using vaginal/genital health and hygiene products both on the outer genital area (externally) and in the vagina (internally). Products were more likely to be used externally than internally, with the exception of some products such as suppositories and douches that are intended for internal use. Over 90% of respondents had ever used (lifetime use) one or more products externally, while 64% reported using one or more products internally.

**Table 2:** Types and prevalence of vaginal health and hygiene product use

Product	Ever used (*N* = 1435)		Used 3 months prior	
	Internal *N* (%)	External *N* (%)	Internal *N* (%)	External *N* (%)
Vaginal moisturizers/lubricants	583 (40.6)	430 (30)	289 (20.1)	220 (15.3)
Vaginal tablets	500 (34.8)	–	43 (3)	–
Anti-itch creams	370 (25.8)	715 (49.8)	44 (3.1)	134 (9.3)
Vaginal wipes	60 (4.2)	602 (42)	37 (2.6)	273 (19)
Vaginal washes/cleansers	58 (4.0)	168 (11.7)	11 (0.8)	66 (4.6)
Baby/antiseptic wipes	37 (2.6)	597 (41.6)	17 (1.2)	260 (18.1)
Hand/body lotion	28 (2.0)	304 (21.2)	6 (0.4)	170 (11.8)
Baby oil	28 (2.0)	149 (10.4)	5 (0.3)	37 (2.6)
Vaginal deodorant suppositories	20 (1.4)	–	4 (0.3)	–
Liquid/gel sanitizers	12 (0.8)	26 (1.8)	2 (0.2)	11 (0.8)
Vaginal sprays	8 (0.6)	81 (5.6)	3 (0.3)	14 (1.0)
Vaginal powders	8 (0.6)	78 (5.4)	4 (0.3)	30 (2.1)
Shaving cream	–	719 (50.1)	–	361 (25.2)
Other (listed in text box)

**Table 3:** Types and prevalence of vaginal practices

Vaginal health and hygiene practice	Ever used (*N* = 1435) *N* (%)
Insertion of sex toys	562 (39.2)
Waxing genital area	380 (26.4)
Genital surgery (reasons other than cosmetic)	34 (2.4)
Genital piercing	24 (1.7)
Traditional genital cutting	20 (1.4)
Pubic hair colouring	15 (1.0)
Genital cutting (for reasons other than traditional)	13 (0.9)
Genital tattoo	12 (0.8)
Anal bleaching	7 (0.5)
Vajazzling (the application of stick-on gemstones to the genital area)	6 (0.4)
Vaginal bleaching	6 (0.4)
Genital cosmetic surgery	6 (0.4)
Smoking/fogging/steaming to tighten vagina	5 (0.3)
Injection to enhance G-spot	4 (0.3)
Other Practices (from text box)	89 (6.2)
Episiotomy/stitches for tear during childbirth	21 (1.4)
Other medical procedure	18 (1.3)
Shaving	13 (0.9)
Laser hair removal	9 (0.6)
Traditional labia stretching	1 (0.07)

Commercially manufactured and advertised products specified as “feminine” or for vaginal health and/or hygiene included douches, wipes, washes, sprays, powders, moisturizers/lubricants, deodorant suppositories, tablet suppositories (e.g., probiotics, tablets or ovules for vaginal infections) and anti-itch creams. Products used in the genital area but not intended or marketed for such use included hand sanitizers, body lotion, baby oil, baby wipes, and shaving cream. With the exception of douches, which included both commercially manufactured and homemade douches, products used by respondents were mostly commercially manufactured. Survey respondents (21.3%) reported using a variety of different commercially manufactured and homemade vaginal douches. Of those participants, 19.6% (*n* = 45) had used at least one type of homemade or commercially manufactured douche in the 6 months prior to completing the survey. Participants also provided information about other products used in the vaginal or genital area not otherwise captured by the survey. Fifteen percent indicated they had used other products, and half of those participants (49.8%) had used the product in the last 3 months. The most common of these products included medicated creams, sprays, and gels (2.7%), cooking oils (1.6%), yogurt (topical and suppositories) (1.4%), garlic clove suppositories (1.1%), probiotic suppositories (0.8%), prescription suppositories (0.08%), depilatory creams (0.8%), and petroleum jelly (0.7%).

Frequency of use ranged considerably across the different products, with a sizable portion of participants using a product in or around the vaginal area at least once per day. For example, among participants who reported using wipes externally in the past 3 months (*N* = 271), 30% used the product at least once a day. A similar percentage of participants (*N* = 37, 29.7%) reported using wipes internally at least once per day in the past 3 months. Similarly high rates of daily use were found with several other products. For example, 46.7% of participants who reported using powders externally (*N* = 30), 35.4% of participants who reported using washes externally (*N* = 65), and 18.4% of participants who reported using baby wipes externally (*N* = 256) reported doing so at least once per day in the past 3 months. There were also several products, including deodorant suppositories and internal use of hand creams, baby oil, and gel sanitizers, that were not used daily by any participants.

Practices related to vaginal/genital health and hygiene included sexual practices (e.g., inserting sex toys into the vagina, G-spot injections), aesthetic practices (e.g., cosmetic surgery, waxing pubic hair, vaginal bleaching), and cultural/religious practices (e.g., traditional genital cutting).

### Associations between product use and adverse health conditions

In this section, we examine the associations between participants’ use of vaginal/genital health and hygiene products and adverse vaginal/genital health conditions. Participants who reported use of any vaginal/genital health and hygiene product(s) had approximately three times higher odds of reporting any adverse health condition (reported history of BV, yeast infection, UTI, or STI) (OR = 3.2, 95% CI: 2.4-4.2) (*p* < .01). Univariate and multivariate analyses to test for confounding demographic factors are presented in Table 4. Participant age and sexual orientation were added to the model, but had no significant effect on the relationship between product use and health condition (OR = 3.2, 95% CI: 4.5-4.3) (*p* < .01).

**Table 4:** Univariate and multivariate logistic regression analysis of any type of adverse health condition

Predictor variables	Univariate		Multivariate	
	OR (CI 95%)	*p*	OR (CI 95%)	*p*
Any product use	3.2 (2.4-4.2)	.00^**^	3.2 (4.5-4.3)	*.*00^**^
Ethnicity		.76		
White	*reference*			
Black	1.3 (0.5 – 3.4)			
Chinese	0.8 (0.3 – 2.7)			
East Asian	0.3 (0.1 – 2.1)			
South Asian	0.5 (0.2 – 2.0)			
Latin/S/Cen American	0.8 (0.1 – 6.8)			
Aboriginal	0.4 (0.6 – 4.1)			
Middle Eastern	0.08 (0.9 – 2.9)			
Mixed	0.8 (0.3 – 2.7)			
Other	0.9 (0.3 – 3.4)			
Education Level		.21		
Less than high school	0.6 (0.2 – 2.3)			
High school diploma	0.7 (0.4 – 1.0)			
Some post-secondary	1.1 (0.7 – 1.5)			
Diploma complete	0.8 (0.5 – 1.2)			
Degree complete	1.0 (0.7 – 1.4)			
Post-graduate complete	*reference*			
Age		.00^**^		.00^**^
Less than 25 years	1.5 (0.4 - 5.5)		1.4 (0.4 – 5.3)	
26-35 years	1.5 (0.4 – 5.6)		1.2 (0.3 – 4.6)	
36-45 years	1.4 (0.4 – 5.2)		1.1 (0.3 – 4.2)	
46-55 years	0.9 (0.2 – 3.2)		0.8 (0.2 – 3.0)	
56- 65 years	0.6 (0.1 – 0.3)		0.5 (0.1 – 2.1)	
66- 75 years	0.2 (0.1 – 2.7)		0.6 (0.1 – 2.7)	
76+ years	*reference*			
Sexual Orientation		.00^**^		.01^*^
Heterosexual	*reference*		1.1 (0.5 – 2.4)	
Lesbian	1.3 (0.6 – 2.7)		1.3 (0.5 – 3.6)	
Bisexual	1.3 (0.5 – 3.4)		2.3 (1.5 – 3.6)	
Questioning/Uncertain	2.7 (1.7 – 4.1)		1.2 (0.6 – 2.5)	
Other	1.4 (0.7 – 2.8)			

Dependent variable: Any adverse health condition

^**^ *p* < .01

^*^ *p* < .05

Several significant associations were found between the use of specific vaginal/genital health and hygiene products (anti-itch creams, moisturizers/lubricants, gel sanitizers, feminine wipes, baby wipes, feminine washes/gels, and douches) and a previous diagnosis of either BV, yeast infection, or UTI. Participants who reported using anti-itch cream had almost 18 times higher odds of reporting a yeast infection (OR = 17.8, 95% CI: 11.9-26.5) (*p* < .01), 5 times higher odds of reporting BV (OR = 4.8, 95% CI: 2.1-10. 8) (*p* < .01), and two times higher odds of reporting a UTI (OR = 2.2, 95% CI: 1.4-3.5) (*p* < .01) than participants who had not used anti-itch creams. Participants who reported using moisturizers/lubricants had 2.5 times higher odds of reporting a yeast infection (OR = 2.5, 95% CI: 1.8-3.5) (*p* < .01) and 50% higher odds of reporting a UTI (OR = 1.5, 95% CI: 1.0-2.1) (*p* = .03) than participants who had not used moisturizers/lubricants. Participants who reported using gel sanitizers had almost 8 times higher odds of reporting a yeast infection (OR = 7.61, 95% CI: 2.3-25.2) (*p* < .01) and almost 20 times higher odds of reporting BV (OR = 19.5, 95% CI: 4.9-77.9) (*p* < .01) than participants who had not used gel sanitizers. Participants who reported using feminine wipes had almost double the odds of reporting a UTI (OR = 1.9, 95% CI: 1.3-2.7) (*p* <. 01). Similarly, participants who reported using baby wipes had almost 60% higher odds of reporting a UTI (OR = 1.6, 95% CI: 1.1-2.3) (*p* = .02). Participants who reported using feminine washes/gels had almost 3.5 times higher odds of reporting BV (OR = 3.4, 95% CI: 1.2-10.1) (*p* = .03) and almost 2.5 times higher odds of reporting a UTI (OR = 2.4, 95% CI: 1.4-4.3) (*p* < .01). Finally, participants who reported using a douche in the previous 6 months had almost 3 times higher odds of reporting a yeast infection (OR = 2.9, 95% CI: 1.5-5.6) (*p* < .01), 7 times higher odds of reporting BV (OR = 7.0, 95% CI: 2.3-22.0) (*p* < .01), and more than 2.5 times higher odds of reporting a UTI (OR = 2.6, 95% CI: 1.3-5.2) (*p* < .01) than participants who had not douched.

Adverse health conditions were not significantly associated with the use of deodorant sprays, powders, baby oils, hand/body creams, or deodorant suppositories. No significant associations were found between having an STI and the use of any particular vaginal/genital health and hygiene product.

## Discussion

The results of this cross-sectional survey identified the high prevalence of particular vaginal/genital health and hygiene behaviors among individuals living in Canada, and identified the most commonly used commercially manufactured and homemade products and practices, including anti-itch creams, feminine wipes, feminine washes/gels, douches, baby wipes, moisturizers/lubricants, tablet suppositories, and pubic hair removal practices. While douching has been the focus of previous research, and douching prevalence in the current study was consistent with national surveys in the US [11, 18] (around 21% of the sample), the prevalence of other products in the current study, particularly anti-itch creams, moisturizers/lubricants, feminine wipes, and baby wipes in and around the vaginal area, was higher than douching. This is consistent with the proliferation of a range of different commercially manufactured products for vaginal/genital health and hygiene now available in most drug and grocery stores.

With respect to symptom prevalence, most participants had experienced at least one vaginal/genital symptom in their lifetime; the most common of which were itching, burning, and unusual discharge. Approximately half of the sample reported experiencing adverse vaginal/genital health conditions such as yeast infection, BV, and UTI. Finally, several notable associations were found between the use of particular vaginal/genital health and hygiene products and various adverse vaginal/genital health conditions.

A better understanding of the types and prevalence of vaginal/genital health and hygiene behaviors is a necessary first step toward understanding the relationships between these behaviors and adverse health conditions, and ultimately their role in the health of the vaginal microbiome. This is particularly important given established connections between vaginal conditions such as BV and other serious sexual health conditions such as HIV and other STIs [25–27] and adverse pregnancy outcomes such as preterm birth and endometriosis [28–30].

While we are unable to make claims about the causal direction of these relationships, our study shows that participants who had used any vaginal/genital health and hygiene product(s) in the 3 months prior had approximately three times higher odds of reporting any adverse health condition, controlling for age and sexual orientation. We also identified several key associations between specific products and adverse health conditions to be more fully explored in future research. While some associations were expected, such as the use of anti-itch creams in relation to yeast infections (a common symptom of which is genital itch), other identified associations point to important areas for future research. Most notably, the use of gel sanitizers was associated with higher odds of having a yeast infection and BV, the use of both feminine and baby wipes was associated with higher odds of UTI, and vaginal moisturizers/lubricants was associated with higher odds of both yeast infection and UTI. Additionally, and consistent with previous research [12–17], douching was associated with higher odds of yeast infection, BV, and UTI.

There are several explanations for the relationships between product use and adverse health conditions that are worth considering. In some cases, women may be using certain products to address symptoms or health conditions. In light of the high rates of reported symptoms, participants already experiencing gynecological conditions may be seeking out over-the-counter or homemade remedies for symptoms management or cessation. Women who, for example, suspect a yeast infection may be seeking over the counter anti-itch creams to manage the condition. Alternatively, vaginal/genital symptoms and health conditions may present as a result of using certain products. As one example, our study found that participants who used gel sanitizers had almost 8 times higher odds of reporting a yeast infection diagnosis than those participants who had not used gel sanitizers. It is possible that gel sanitizers, which often include dyes and other chemical ingredients, cause or exacerbate the yeast infection through disruption of the natural vaginal microbiome or through micro-abrasions caused inside the vagina. It will be important for future scientific and medical research to explore the associations identified in the current study because determining the specific nature of the relationship is critical for informing clinical practice. Regardless of the direction of the relationship between product use and health conditions, education and outreach about product efficacy and safety is necessary.

### Strengths and limitations

This is the first study to collect comprehensive data on diverse vaginal/genital health and hygiene behavior among respondents living in Canada. Previous research examining vaginal/genital practices and their impact on vaginal ecology or health outcomes has narrowly focused on specific behaviors, such as douching. Our aim was to recruit a large Canadian sample that resembled national demographic statistics within a reasonable timeframe.

While our sample was close to resembling national statistics across several key demographics, including ethnicity, sexual orientation, and education (e.g., 89% White in sample compared to 83% nationally), we were not as successful as we had intended in the number of participants we recruited residing in Quebec and those 40 years and older. Despite this limitation, this research provides the first account of vaginal/genital health and hygiene behaviors at the national level. Although these findings may generalize to individuals living in Canada, further scientific research with more complex statistical analysis is necessary.

In future research, temporal periods for engaging in vaginal/genital health and hygiene behaviors and incidence of symptoms and health conditions should be consistent to ensure meaningful interpretation of analysis. Additional information, such as menopausal status and the use of hormonal therapies, would be of further benefit. Finally, given the exploratory nature of these data and the small number of participants who reported using certain products, our analysis was limited to description and regression analysis and as such causal relationships cannot be inferred. Although several of the effect sizes for particular associations seemed quite large, it was difficult to get a precise estimate due to small cell counts in some cases and these should be interpreted with caution. Future research using prospective or case control designs can build on these preliminary correlational findings to assess causal links between vaginal/genital health and hygiene behaviors and vaginal/genital symptoms and adverse health conditions.

## Conclusions

The purpose of this exploratory study was to identify the types and prevalence of vaginal/genital symptoms, health conditions, and health and hygiene behaviors among Canadians. This research can inform medical researchers and practitioners about the diversity and prevalence of vaginal symptoms, adverse gynecological health conditions, and importantly, vaginal/genital health behaviors that may be relevant to abnormal microbial profiles as microbiome research advances. Future biological research can extend the current study by identifying causal relationships between vaginal health and hygiene behaviors and changes to the vaginal microbiome.

Our findings will help inform healthcare professionals and the public about possible areas of concern regarding products and practices. Previous research has identified a link between douches and vaginal infections, and as this and other relationships between health and hygiene behaviors and adverse gynecological outcomes are further examined, it will be important for healthcare providers, public health units, and governments to respond accordingly to inform the public and put in place necessary warning labels and restrictions. Our study shows that a large number of respondents use products inside the vagina even when the product is not intended for use in the genital/vaginal area or product labels warn against internal use (e.g., vaginal washes and wipes). Should future research find some products to be safe and effective for addressing vaginal symptoms and health conditions, healthcare providers will similarly want to discuss this with patients. The increasing availability and variety of vaginal health and hygiene products requires oversight and regulation regardless of the causal nature of the relationship between products and health conditions. Evidence-based information about the safety and efficacy of these products is paramount to ensuring women’s health. This information can assist women in making informed choices when choosing products or partaking in various health, social, and/or cultural practices.

## Abbreviations

BV: Bacterial vaginosis
HPV: Human papillomavirus
STI: Sexually transmitted infection
UIT: Urinary tract infection

## References

[1] MacPhee RA, Hummelen R, Bisanz JE, Miller JE, Reid G (2010). Probiotic strategies for treatment and prevention of bacterial vaginosis. Expert Opin Pharmacother.

[2] Hummelen R, Macklaim JM, Bisanz JE, Hammond J, McMillan A, Vongsa R (2011). Vaginal microbiome and epithelial gene array in post-menopausal women with moderate to severe dryness. PLoS One.

[3] Ravel J, Gajer P, Abdo Z, Schneider GM, Koenig S, Mcculle SL (2011). Vaginal microbiome of reproductive-age women. P Natl. Acad Sci.

[4] Chaban B, Links MG, Jayaprakash TP, Wagner EC, Bourque DK, Lohn Z (2014). Characterization of the vaginal microbiota of healthy Canadian women through the menstrual cycle. Microbiome.

[5] Jayaprakash TP, Schellenberg JJ, Hill JE, Badger JH (2012). Resolution and characterization of distinct cpn60-based subgroups of Gardeneralla vaginalis in the vaginal microbiota. PLoS One.

[6] Hill JE, Goh SH, Money DM, Doyle M, Li A, Crosby WL (2005). Characterization of vaginal microflora of healthy, nonpregnant women by chaperonin-60 sequence-based methods. Am J Obstet Gynecol.

[7] Martin DH (2012). The microbiota of the vagina and its influence on women’s health and disease. Am J Med Sci.

[8] Martin DH, Marrazzo JM (2016). The vaginal microbiome: current understanding and future directions. J Infect Dis.

[9] Hickey RJ, Zhou X, Pierson JD, Ravel J, Forney LJ (2012). Understanding vaginal microbiome complexity from an ecological perspective. Transl Res.

[10] Bradshaw CS, Walker SM, Vodstrcil LA, Bilardi JE, Law M (2014). The influence of behaviors and relationships on the vaginal microbiota of women and their female partners: the WOW health study. J Infect Dis.

[11] Centre for Disease Control and Prevention (2013). Key statistics from the national survey of family growth.

[12] Martin Hilber AM, Francis SC, Cherisch M, Scott P, Redmond S, Bender N (2010). Intravaginal practices, vaginal infections and HIV acquisition: systematic review and meta-analysis. PLoS One.

[13] Holzman C, Leventhal JM, Qui H, Jones NM, Wang J (2001). Factors linked to bacterial vaginosis in nonpregnant women. Am J Pub Health.

[14] Fiscella K, Franks P, Kendrick JS, Meldrum S, Kieke BA (2002). Risk of preterm birth that is associated with vaginal douching. Am J Obstet Gynecol.

[15] Zhang J, Thomas AG, Leybovich E (1997). Vaginal douching and adverse health effects: a metaanalysis. Am J Pub Health.

[16] Baird DD, Weinberg CR, Voigt LF, Daling JR (1996). Vaginal douching and reduced fertility. Am J Pub Health.

[17] Brotman RM, Klebanoff MA, Nansel TR, Andrews WW, Schwebke JR (2008). A longitudinal study of vaginal douching and bacterial vaginosis: a marginal structural modelling analysis. Am J Epidemiol.

[18] Koumans EH, Sternberg M, Bruce C, McQuillan G, Kendrick J (2007). The prevalence of bacterial vaginosis in the United States, 2001-2004; associations with symptoms, sexual behaviors, and reproductive health. Sex Trans Dis.

[19] Grimely DM, Annang L, Foushee HR (2006). Vaginal douches and other feminine hygiene products: Women’s practices and perceptions of product safety. Matern Child Healt J.

[20] Czerwinski BS (1996). Adult feminine hygiene practices. Appl Nurs Res.

[21] Ott MA, Ofner S, Fortenberry JD (2009). Beyond douching: use of feminine hygiene products and STI risk among young women. J Sex Med.

[22] François I, Bagnol B, Chersich M, Mbofana F, Mariano E, Nzwalo H (2012). Prevalence and motivations of vaginal practices in Tete Province, Mozambique. Int J Sex Healt.

[23] Martin Hilber A, Hull TH, Preston-Whyte E, Bagnol B, Smit J, Wacharasin C (2009). A cross cultural study of vaginal practices and sexuality: implications for sexual health. Soc Sci Med.

[24] IBM Corp (2016). IBM SPSS statistics for Mac, version 24.0.

[25] Brotman RM, Klebanoff MA, Nansel TR, Yu KF, Andrews WW (2010). Bacterial vaginosis assessed by gram stain and diminished colonization resistance to incident gonococcal, chlamydial, and trichomonal genital infection. J Infect Dis.

[26] Martin HL, Richardson BA, Nyange PM, Lavreys L, Hillier SL (1999). Vaginal lactobacilli, microbial flora, and risk of human immunodeficiency virus type 1 and sexually transmitted disease acquisition. J Infect Dis.

[27] Taha TE, Hoover DR, Dallabetta GA, Kumwenda NI, Mtimavalye LA (1998). Bacterial vaginosis and disturbances of vaginal flora: association with increased acquisition of HIV. AIDS.

[28] Leitich H, Bodner-Adler B, Brunbauer M, Kaider A, Egarter C (2003). Bacterial vaginosis as a risk factor for preterm delivery: a meta-analysis. Am J Obstet Gynecol.

[29] Hillier SL, Kiviat NB, Hawes SE, Hasselquist MB, Hanssen PW (1996). Role of bacterial vaginosis-associated microorganisms in endometritis. Am J Obstet Gynecol.

[30] Hillier SL, Nugent RP, Eschenbach DA, Krohn MA, Gibbs RS (1995). Association between bacterial vaginosis and preterm delivery of a low-birth-weight infant. The vaginal infections and prematurity study group. N Engl J Med.

